# What Organization May Accomplish

**Published:** 1916-03

**Authors:** 


### What Organization May Accomplish.

Every organization should have definite purposes in view and should work to their accomplishment. Most of them do have an outlined plan of work, but with many associations the outline is about all that is ever seen. The result is that such associations finally resolve themselves into mere social events and mutual admiration societies, where the most learned men display their erudition for the enlightenment of the less favored and the most popular are boosted into the offices. Occasionally an association is so pleased with the "good times" it has at its annual fiestas that it allows itself to build up a strong machine, financial, political, or otherwise, and then it drags along as the puppet of some shrewd financier who profits by its indifference.

The Texas Medical Association is composed of men about like men in other professions, with about the same good impulses and the same weaknesses. It is so easy to drift along from one year to another, enjoying pleasureable associations of the yearly meetings, reading and hearing read papers, passing harmless and helpless resolutions, but in reality accomplishing little that is worth while. It is the constant temptation to which most people are subjected in their lives—to drift with the tide, because it requires no resistance. Organized medicine in Texas has done some notable things that have entitled it to more than ordinary distinction, but it should not be content to rest with what it has accomplished.

Medicine has a humanitarian side to it that needs cultivation. Men are and should be practicing the profession to make a living for themselves and those dependent on them, but above and beyond that there is the idea of service to others that should actuate every human being. Physicians, because of their superior education and knowledge, their confidential relation to the public, their leadership, should be foremost in doing good.

Especially should they be concerned about those who are rendered unfortunate through suffering and disease. This should not be simply a wish that the conditions might be improved, but it should be such a vital concern that will prompt every physician to do his utmost to improve the conditions that result in such disease. What they cannot do individually they can accomplish as an organization, and there is no agency that is in better position than the Texas Medical Association for making itself useful.

The Association should be a strong ally of the State Health Department in its efforts to avoid and suppress contagions and epidemics. It should co-operate with the Pure Food Department in seeing that pure and wholesome foods are supplied the people. It should work persistently with the sanitary officers to promote educational campaigns for personal and public cleanliness. It should see that places for breeding flies and mosquitoes are eliminated and that the water supply is everywhere pure and wholesome. It should demand that proper health conditions exist in jails and penitentiaries, remembering that virtue can not long last in filth and vermin. It should know that the insane asylums—the homes of our most unfortunate class—are officered by men of ability, who have at heart the welfare of the inmates and who see that their surroundings are conducive to comfort and to health.

Should the Association in Texas lend itself actively, even at considerable expense, to looking after such things as are here suggested, aggressively undertaking to remedying existing evils where they touch the domain of medicine and public health, it would become the most powerful and most popular organization in the State; its influence would be without limit; it would have but to make its wants known to have them met by the Legislature.

There is so much creative work to be done in Texas that it seems a pitiful waste of energy, as well as of organization, that more really creditable things are not undertaken by the membership of the Texas Medical Association. It should never be satisfied to rest on its past achievements, but, encouraged by what it has accomplished, it should strive for still greater things.

### Average Pay is Poor.

Dr. Otto V. Huffman, Secretary of the New York State Board of Health, is authority for the statement that physicians average only about $500 a year. With this small compensation there is little inducement for our brainy young men to spend time and money in a medical education. Still the fact that thousands of physicians make munificent sums will continue to tempt the mediocre men to enter the profession. There is little danger that the announcement of the small annual pay received will keep down the number of physicians.

### Leprosy is Spreading.

The health authorities at Washington are in receipt of what appears to be correct information that there are more than 500 victims of leprosy at liberty in this country, about fifty of which are in New York. It is declared that the disease is spreading and that a serious epidemic is threatened unless the government takes active steps to check it. The Rockefeller Foundation thinks it has found a remedy that, while it is not claimed as a specific, gives more consistently favorable results than any other known treatment, and offers hope that a specific may yet be found. The remedy being tried out by the Rockefeller Foundation is Chaulmoogra oil, made from the seeds of a plant found in the Far East, the oil being administered hypodermically, sometimes in combination with camphorated olive oil.

